# The hemostatic and anti-inflammatory effects of intravenous single-dose of tranexamic acid in double-segment posterior lumbar interbody fusion: a case control study

**DOI:** 10.1038/s41598-024-62823-4

**Published:** 2024-06-04

**Authors:** Shenshen Hao, Shiying Luo, Zhan Zhao, Shengli Dong, Shuai Liu, Hongke Li, Binbin Li, Xinhao Cao

**Affiliations:** 1 Department of Spine and Bone Oncology, General Hospital of Pingmei Shenma Medical Group, Pingdingshan City, Henan Province China; 2 Office of the Ombudsman, Pingdingshan University, Pingdingshan City, Henan Province China; 3 Clinical Research and Teaching Center, General Hospital of Pingmei Shenma Medical Group, Pingdingshan City, Henan Province China; 4 Department of Rehabilitation Medicine, Haikou Hospital Affiliated to Xiangya Medical College of Central South University, Haikou City, Hainan Province China; 5 Emergency Medicine Department of Anesthesia Emergency and Critical Care Diagnosis and Treatment Center, Honghui Hospital Xi’an Jiaotong University, No. 555, Youyi East Road, Nanshaomen, Beilin District, Xi’an City, 710000 Shaanxi Province China

**Keywords:** Double-segment posterior lumbar interbody fusion, Single-dose intravenous administration, Tranexamic acid, Blood loss, C-reactive protein, Erythrocyte sedimentation rate, Adaptive clinical trial, Outcomes research

## Abstract

This study aims to observe the hemostatic and anti-inflammatory effects of intravenous administration of tranexamic acid (TXA) in dual segment posterior lumbar interbody fusion (PLIF). The data of 53 patients with lumbar disease treated with double-segment PLIF were included in this study. The observation group was received a single-dose intravenous of TXA (1 g/100 mL) 15 min before skin incision after general anesthesia. The control group was not received TXA. The observation indicators included postoperative activated partial prothrombin time (APTT), thrombin time (PT), thrombin time (TT), fibrinogen (FIB), platelets (PLT), and postoperative deep vein thrombosis in the lower limbs, surgical time, intraoperative bleeding volume, postoperative drainage volume, transfusion rate, postoperative hospital stay, red blood cell (RBC), hemoglobin (HB), hematocrit (HCT), C-reactive protein (CRP), and erythrocyte sedimentation rate (ESR) on the 1st, 4th, 7th, and last tested day after surgery. All patients successfully completed the operation, and there was no deep vein thrombosis after operation. There was no statistically significant difference in postoperative APTT, PT, TT, FIB, PLT, surgical time, and postoperative hospital stay between the two groups (p > 0.05). The intraoperative bleeding volume, postoperative drainage volume, and transfusion rate in the observation group were lower than those in the control group, and the differences were statistically significant (p < 0.05). There was no statistically significant difference in RBC, HB, HCT, CRP, and ESR between the two groups on the 1st, 4th, 7th, and last tested day after surgery (p > 0.05). Intravenous administration of TXA in dual segment PLIF does not affect coagulation function and can reduce bleeding volume, postoperative drainage volume, and transfusion rate. Moreover, it does not affect the postoperative inflammatory response.

## Introduction

Posterior lumbar interbody fusion (PLIF) is currently one of the classic surgical methods for the treatment of lumbar diseases, such as lumbar disc herniation (LDH), lumbar spinal stenosis (LSS), and lumbar spondylolisthesis (LS)^1,2^. However, PLIF has a problem of large perioperative blood loss^3,4^. A previous literature had shown that the total loss of blood in dual segment PLIF could reach over 1000 mL^5^. Tranexamic acid (TXA), trans-4-aminomethylcyclohexanecarboxylic acid, was discovered in 1962 as a synthetic derivative of lysine^6^. As a plasmin inhibitor, it can inhibit the plasminogen activator by competitively inhibiting the activation of plasminogen to plasmin and play an anti-fibrinolytic effect to achieve hemostasis^6^. Many scholars have reported that intravenous TXA can reduce perioperative blood loss in PLIF^7–9^. The 2019 Chinese expert consensus recommended intravenous methods include single, continuous and multiple method^10^. Among them, intravenous of 1 g of TXA 15 min before skin incision is a typical single-dose method with a good anti-fibrinolytic effect. Besides, this application is simple to operate without interfering with intraoperative fluid, and can avoid adverse reactions between various drugs that may occur during anesthesia^11^.

Meanwhile, PLIF has a high risk of postoperative infection. In recent years, more and more studies have shown that in addition to the anti-fibrinolytic effect of TXA, its anti-inflammatory effect also has important clinical significance^12,13^. C-reactive protein (CRP) and erythrocyte sedimentation rate (ESR) are commonly used inflammatory indicators for diagnosing infection. As inflammation monitoring indexes, the specificity of CRP is 90.27%, and the sensitivity of ESR is 88.50%^14^. One study showed that the sensitivity of CRP and ESR in diagnosis of deep wound infection was significantly higher than those of infection-related indicators such as body temperature, local physical signs of wound, white blood cell count and neutrophil count^15^. However, the changes of CRP and ESR in the perioperative period of PLIF are rarely observed. In our previous study, we observed the changes of CRP and ESR during the perioperative period of single-segment PLIF with preoperative intravenous TXA^16^. It was unknown whether the application of single-dose TXA will affect the CRP and ESR in double-segment PLIF.

Therefore, the purpose of this study was twofold. One was to observe the hemostatic effect of intravenous method of TXA in dual segment PLIF. The second was its anti-inflammatory effect.

## Method

### Object of research

The data of patients with lumbar disease treated with PLIF from 2020.10 to 2023.4 were retrospectively collected. The inclusion criteria included a. The preoperative diagnosis was LDH, LSS and LS, and conservative treatment was ineffective. b. The American Society of Anesthesiologists rated it as Level 2. c. The scope of operation was two-segment. d. The medical records meeting the research requirements were complete. Exclusion criteria included a. There was infectious disease before the operation. b. There were blood system diseases before operation. c. There was a history of lumbar surgery. d. There was a lumbar fracture. e. There was deep venous thrombosis (DVT) of lower limbs before operation. f. There was a history of smoking or diabetes mellitus. g. Cerebrospinal fluid leakage or dural injury occurred during operation. A total of 53 cases were included in this study, including 20 males and 33 females, aged 34–80 years, with an average of (59.736 ± 11.339) years. The patients agreed to the surgery and the study was approved by the Ethics Committee of the General Hospital of Pingmei Shenma Medical Group (No. 2021004). According to whether the single-dose intravenous TXA was administered or not, they were divided into an observation group of 27 cases and a control group of 26 cases.

$$N=\frac{{2({z}_{\alpha }+{z}_{\beta } )}^{2}{\sigma }^{2}}{{d}^{2}}$$

The calculation of sample size (N) comes from the above formula. N is the sample size, σ is the estimated standard deviation of two populations, and d is the difference between the means of two groups, α = 0.05, β = 0.2. This study conducted a double-sided test, and the table showed that ${z}_{\alpha }=1.96$, ${z}_{\beta }=0.84$. According to the results of prothrombin time (PT) in previous literature, the control group was 13.34 ± 0.99 and the observation group was 12.34 ± 0.79 after treatment. Based on the above, it is estimated that ${\sigma }^{2}=1.60$, calculated as 25 people per group, considering a 5% dropout rate, it is 26 people, which means the minimum sample size for each group is 26 people. A total of 53 people were included in this study, meeting the research needs.

The demographic data included age, gender, body mass index (BMI), disease type, activated partial thrombin time (APTT), PT, thrombin time(TT), fibrinogen (FIB), platelets (PLT), red blood cell (RBC), hemoglobin (HB), hematocrit (HCT), CRP, and ESR. There was no significant difference in the demographic data between the two groups (p > 0.05) (Table 1).

**Table 1:** Comparison of the demographic data of the two groups.

Groups	Observation group (n = 27)	Control group (n = 26)	t/χ^2^/Z value	p value
Age (year)	61.370 ± 9.022	58.038 ± 13.301	1.071	0.289
Gender, n			0.212	0.646
Male	11	9		
Female	16	17		
BMI (kg/m^2^)	24.002 ± 2.952	25.304 ± 2.385	− 1.762	0.084
Disease type, n			2.525	0.283
LDH	3 (11)	7		
LSS	17 (63)	15		
LS	7 (26)	4		
HB (g/L)	134.074 ± 13.041	134.769 ± 15.122	− 0.179	0.858
RBC (10^12^/L)	4.31 [4.08; 4.54]	4.26 [4.17; 4.49]	− 0.267	0.789
HCT (L/L)	0.40 [0.37; 0.42]	0.40 [0.38; 0.42]	− 0.455	0.649
APTT (s)	30.5 [29.8; 33.0]	32.0 [29.1; 33.1]	− 0.062	0.950
PT (s)	11.485 ± 0.726	11.192 ± 0.863	1.339	0.187
TT (s)	14.525 ± 1.107	14.779 ± 0.966	− 0.886	0.380
FIB (g/L)	2.819 ± 0.501	2.771 ± 0.410	0.382	0.704
PLT (10^9^/L)	192 [164; 235]	235 [172; 281]	− 1.281	0.200
CRP (mg/L)	0 [0; 0.58]	0.72[0; 1.69]	− 1.848	0.065
ESR (mm/h)	14.926 ± 9.450	15.269 ± 9.986	− 0.129	0.898

### Research method

All patients were under general anesthesia. The observation group was given a single administration method, that was, intravenous single-dose of TXA (1 g/100 mL) was used 15 min before skin incision after general anesthesia. The control group was not given TXA. The surgical positions of the two groups were all in the prone position, and the operations were the same, both of which were standard double-segment PLIF. Two negative pressure drainage tubes were placed postoperatively and were removed when the volume was less than 50 mL/24 h. They received similar routine treatment measures after operation, such as antibiotics, glucocorticoids, dehydration, anticoagulation, nutritional support, wound dressing change, and monitoring of DVT of lower limbs.

### Data collection

The observation indicators included postoperative APTT, PT, TT, FIB, PLT, and DVT in the lower limbs, surgical time, intraoperative bleeding volume, postoperative drainage volume, transfusion rate, postoperative hospital day, RBC, HB, HCT, CRP, and ESR on the 1st, 4th, 7th, and last tested day after surgery.

### Statistical analysis

Data analysis was performed via SPSS statistical software, version 22.0. If the data follows a normal distribution, it is expressed as mean ± standard deviation, and t-test is used for comparison between the two groups. Otherwise, use M [P25; P75] to represent and compare between the two groups using Mann Whitney U test. Count data is compared between groups using chi square test. P < 0.05 indicates a statistical difference. The trend of changes in CRP and ESR during the perioperative period, represented by the median, was plotted using an Excel table.

### Ethics approval and consent to participate

This study had been performed in accordance with the Declaration of Helsinki, and was approved by the Ethics Committee of the General Hospital of Pingmei Shenma Medical Group, and the reference number was 2021004. All authors confirmed that informed consent was obtained from all subjects.

## Result

All patients successfully completed the operation. There was no DVT occurred after the surgery. There was no significant difference in postoperative APTT, PT, TT, FIB and PLT between the two groups (p > 0.05) (Table 2).

**Table 2:** Comparison of the postoperative coagulation indicators between the two groups.

Groups	Observation group (n = 27)	Control group (n = 26)	t/χ^2^/Z value	p value
APTT (s)	28.481 ± 1.921	28.242 ± 2.597	0.382	0.704
PT (s)	12.4 [11.8; 12.9]	11.9 [11.5; 12.4]	− 1.576	0.115
TT (s)	14.10 [13.40; 14.90]	14.20 [14.00; 14.88]	− 1.121	0.262
FIB (g/L)	2.92 [2.62; 3.11]	2.73 [2.41; 3.06]	− 1.486	0.137
PLT (10^9^/L)	172 [160; 214]	207 [165; 228]	− 1.424	0.155

There was no statistically significant difference in surgical time and postoperative hospital stay of the two groups (p > 0.05). The intraoperative bleeding volume, postoperative drainage volume, and transfusion rate in the observation group were lower than those in the control group, and the difference was statistically significant (p < 0.05). There was no significant difference in RBC, HB, and HCT between the two groups on 1st, 4th, 7th, and last tested day after surgery (p > 0.05) (Table 3).

**Table 3:** Comparison of the blood loss related indicators between the two groups.

Groups	Observation group (n = 27)	Control group (n = 26)	t/χ^2^/Z value	p value
Surgical time (min)	203.444 ± 44.599	209.500 ± 47.560	− 0.478	0.634
Intraoperative blood loss (mL)	400 [300; 500]	550 [400; 800]	− 2.429	0.015
Postoperative drainage volume (mL)	266.481 ± 72.614	389.808 ± 100.284	0.700	0.000
Blood transfusion rate, %(n)	7.407	38.462	7.293	0.007
Yes	2	10		
No	25	16		
Postoperative hospital stay (day)	16.148 ± 7.508	13.500 ± 3.050	1.670	0.101
HB (g/L)				
Postoperative 1st day	118.111 ± 12.411	115.769 ± 14.946	0.172	0.537
Postoperative 4th day	111.407 ± 15.280	108.462 ± 15.373	0.700	0.487
Postoperative 7th day	112 [105; 126]	108 [104; 123]	− 1.273	0.203
Last tested day	116.037 ± 10.801	113.846 ± 14.153	0.635	0.528
RBC (10^12^/L)				
Postoperative 1st day	3.756 ± 0.408	3.722 ± 0.460	0.285	0.777
Postoperative 4th day	3.46 [3.17; 4.07]	3.41 [3.19;3.71]	− 0.721	0.471
Postoperative 7th day	3.59 [3.39; 4.07]	3.40 [3.32; 3.90]	− 1.263	0.206
Last tested day	3.712 ± 0.338	3.687 ± 0.442	0.238	0.813
HCT (L/L)				
Postoperative 1st day	0.346 ± 0.035	0.341 ± 0.042	0.415	0.680
Postoperative 4th day	0.32 [0.29; 0.34]	0.31 [0.29; 0.34]	− 537	0.592
Postoperative 7th day	0.33 [0.31; 0.36]	0.32 [0.30; 0.36]	− 0.939	0.348
Last tested day	0.34 [0.32; 0.35]	0.34 [0.31; 0.35]	− 0.260	0.795

**Figure 1:**
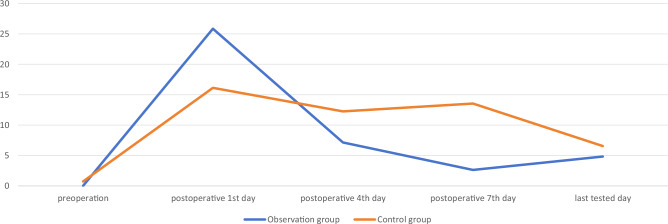
The trend of changes of CRP during the perioperation in the two groups, mg/L.

**Figure 2:**
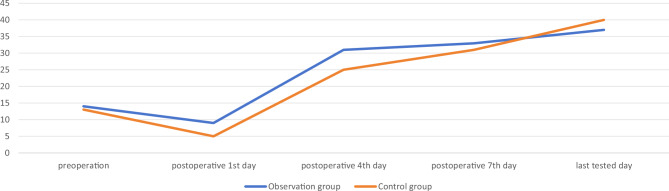
The trend of changes of ESR during the perioperation in the two groups, mm/h.

There was no significant difference in CRP and ESR between the two groups on 1st, 4th, 7th, and last tested day after surgery (p > 0.05). The comparison was shown in Table 4, Figs. 1 and 2.

**Table 4:** Comparison of the inflammatory response indicators between the two groups.

Groups	Observation group (n = 27)	Control group (n = 26)	t/χ^2^/Z value	p value
CRP (mg/L)				
Postoperative 1st day	25.86 [11.12; 32.05]	16.13 [11.73; 27.82]	− 1.521	0.128
Postoperative 4th day	7.12 [0.96; 12.98]	12.26 [5.36; 21.39]	− 1.744	0.081
Postoperative 7th day	2.63 [1.15; 16.41]	13.57 [2.17; 34.60]	− 1.550	0.121
Last tested day	4.83 [0.90; 15.81]	6.55 [1.36; 21.21]	− 0.482	0.630
ESR, mm/h				
Postoperative 1st day	9 [2; 15]	5 [2; 8]	− 1.486	0.137
Postoperative 4th day	29.519 ± 18.554	26.462 ± 17.217	0.621	0.537
Postoperative 7th day	32.296 ± 19.766	41.115 ± 33.281	− 1.178	0.244
Last tested day	37 [23; 54]	40 [24; 49]	− 0.044	0.965

## Discussion

Although there have been many studies on the safety and hemostatic effects of TXA in PLIF. Most scholars reported that intravenous TXA in PLIF could effectively reduce perioperative blood loss without increasing the risk of DVT^17–19^. However, some researchers still believed that TXA could inhibit the dissolution of fibrin, thereby increasing the risk of DVT^20^. Therefore, if studying the intravenous use of TXA in PLIF, its safety had to be considered. In this study, all the operations of groups were successfully completed, and there were no significant abnormalities in postoperative APTT, PT, TT, FIB, and PLT. And there was no occurrence of DVT after surgery. Therefore, this indicated that intravenous TXA was safe and feasible in dual segment PLIF.

In our early study, we found that preoperative intravenous administration of TXA in single segment PLIF could reduce intraoperative bleeding and postoperative drainage^16^. This study found that intravenous application of TXA in dual segment PLFI could also reduce intraoperative bleeding, postoperative drainage, and transfusion rate. This was similar to the research results of Gao et al.^21^. Generally speaking, reducing intraoperative bleeding also reduces the need for intraoperative hemostasis. This will make the field of view of the surgical area clearer, more conducive to surgical operations, and ultimately reduce surgical time. However, different scholars have different results in terms of surgical time. Qiao et al. found that the application of TXA in PLIF could shorten surgical time^22^. This study found that the application of TXA did not reduce the operative time, which was similar to the results of Wang et al.^23^. It was worth noting that different results were obtained in the research of Sun et al.^24^. They applied 1g of TXA intravenously 15 min before the PLIF surgery, which extended the surgical time compared to the control group who did not use TXA. The reasons for the inconsistency in the above surgical time were related to the following points. One was related to the sample size of the study. When the sample size was small, it may inevitably cause bias in the research results. The second was related to the cooperation between surgical techniques and surgical teams. In theory, a decrease in intraoperative bleeding and postoperative drainage volume will maintain high levels of postoperative RBC, HB, and HCT. However, our study yielded different results. There was no significant difference in RBC, HB, and HCT measured at different time after surgery between the two groups. We believed that there might be two reasons for this result. Firstly, although TXA had a hemostatic effect, its half-life was short, and its sustained hemostatic effect was only a few hours. Secondly, although the control group had a large amount of intraoperative bleeding, the transfusion rate was higher. These combined results resulted in the control group maintaining higher levels of RBC, HB, and HCT. Meanwhile, the above might also be reasons for the similar postoperative hospital stay between the two groups.

With the successful application of TXA as a hemostatic in orthopedic surgery in recent years, more and more studies have shown that TXA has potential anti-inflammatory effects^25–28^. Therefore, we observed the anti-inflammatory effect of single dose intravenous injection of TXA on dual segment PLIF. CRP and ESR are commonly used indicators in clinical practice to monitor inflammatory reactions. CRP is an acute phase protein synthesized by hepatocytes with high specificity and sensitivity. When the body is stimulated by inflammation, it can mobilize complement and immune cells to clear pathogens and injured cells^29^. The half-life of CRP is 15 h, and the CRP value of 99% of healthy people is less than 10 mg/L^30^. One study found that CRP could be 10 to 60 times higher than the normal value when it was elevated, and then decreased rapidly, generally 14 days after surgery, to a normal value of 6.76 mg/L^31^. ESR is also one of commonly used clinical indicators to monitor postoperative infection after PLIF^32^. When the body suffers from infection, tissue damage, necrosis, or some disease activity, progression, and deterioration, the plasma resistance of RBC after they overlap each other in a "coin" shape decreases, thereby accelerating ESR^33^. ESR will increase within one week after lumbar spine surgery, and return to the baseline level in two to three weeks, but more than 25 mm/h^34^. A study has shown that combined monitoring of CRP and ESR in spinal surgery conduced to improve the diagnosis rate of postoperative infection^35^.

A study has showed that in orthopedic surgery, the anti-inflammatory effect of TXA was shown by the lower CRP level of patients who received TXA compared with those who not or small dose^36^. However, the specific anti-inflammatory mechanism of TXA is still unclear. Different researchers put forward different opinions according to their research results. Later et al.^37^ studied the mRNA expression level of 114 inflammatory genes before and after operation, and found that TXA could reduce the expression of inflammatory genes. Kong et al.^38^ believed that the anti-inflammatory mechanism of TXA may be that it could protect PLT by reducing the degradation of PLT membrane glycoprotein receptors by plasmin. By protecting PLT, the activation, adhesion and aggregation of PLT can be inhibited, and at the same time, it could effectively inhibit the aggregation of leukocytes and the activation of endothelial cells, so as to reduce the inflammatory reaction. Although fibrinolysis and inflammation are independent processes in many aspects, it is likely that there is a close relationship between fibrinolysis and inflammation system^39^. Among them, fibrinolytic enzyme plays an important role in the process of connecting the fibrinolytic system and the inflammatory system, and it can stimulate the release of cytokines and other inflammatory mediators of different cell types to promote inflammation^40^. In addition, fibrinolytic enzyme can activate the body's complement system by cracking the complement protein while playing the role of thrombolysis. After the activation of the complement system, it can cause and strengthen the inflammatory response through chemotaxis, anaphylaxis, kininoid action and other ways^41^. However, we found that there were three problems, when study the anti-inflammatory mechanism of TXA. First, there were few reports on the anti-inflammatory effects of TXA, and most of them are analyzed around joint surgery. Second, when reviewing previous studies, it was found that the dosage, frequency and timing of TXA were not completely consistent, and these factors might affect the anti-inflammatory effect of TXA. Third, there were many indicators used to display inflammatory reaction, such as interleukin, tumor necrosis factor, CRP and ESR, while different inflammatory factors may reflect different inflammatory reactions. Therefore, the anti-inflammatory mechanism of TXA still needs further study.

In this study, preoperative intravenous administration of 1 g of TXA did not significantly affect the postoperative CRP and ESR. This is similar to our previous results in single-segment PLIF with the same dose and administration method^16^. The research results of Yuan et al.^20^ showed that the postoperative inflammation indicators of patients who received TXA were lower than those of the control group who did not receive TXA, and the improvement of inflammation indicators after multiple used of TXA was better than that of single dose used. Meanwhile, a study has also shown that as the dosage of TXA increases, its anti fibrinolytic effect was enhanced, which might have potential anti-inflammatory effects^42^. However, this study yielded different results, with no significant difference in postoperative CRP and ESR between the two groups. There may be two reasons for the different results mentioned above. On the one hand, we only used 1 g of TXA once before surgery, which belonged to low dose single application, and might not reach the sign of causing detectable anti-inflammatory reaction. Because the anti-inflammatory effect of TXA in the perioperative period had a significant positive correlation with the times and dose of TXA^39^. On the other hand, the use of antibiotics during the perioperative period might mask the anti-inflammatory effect of TXA, which was difficult to be detected.

## Conclusion

In summary, through this study, intravenous infusion of TXA is safe and feasible in dual segment PLIF. It does not affect coagulation function and can reduce intraoperative bleeding, postoperative drainage, and transfusion rate. Moreover, it does not affect the postoperative inflammatory response, especially CRP and ESR. However, this study also has some limitation, as it is a retrospective and relatively small sample size study. In the future, further research will be conducted.

## Data Availability

The datasets used and analyzed during the current study are available from the corresponding author upon reasonable request.

## Abbreviations

TXA: Tranexamic acid
PLIF: Posterior lumbar interbody fusion
APTT: Activated partial thromboplastin time
PT: Prothrombin time
TT: Thrombin time
FIB: Fibrinogen
PLT: Platelets
RBC: Red blood cell
HB: Hemoglobin
HCT: Hematocrit
CRP: C-reactive protein
ESR: Erythrocyte sedimentation rate
BMI: Body mass index
LSS: Lumbar spinal stenosis
LDH: Lumbar disc herniation
LS: Lumbar spondylolisthesis
DVT: Deep venous thrombosis
